# STOX1 Isoform A Promotes Proliferation and Progression of Hepatocellular Carcinoma by Dual Mechanisms of Transcriptionally Upregulation of Cyclin B1 and Activation of ROS‐Dependent PTEN/AKT1 Signaling

**DOI:** 10.1002/cam4.70958

**Published:** 2025-06-26

**Authors:** Chunlin Jiang, Chong Wang, Jian Ao, Yangping Liu, Fengjie Sun, Wangpan Shi, Zeyi Guo, Yanping Wu, Luxiang Gan, Meimei Wu, Yaofeng Zhi, Zijie Meng, Wanting Wu, Juanhua Wu, Yong Ye, Xin Zhang, Dong Ren, Mingxin Pan

**Affiliations:** ^1^ Department of Hepatobiliary Surgery II, Zhujiang Hospital Southern Medical University Guangzhou P. R. China; ^2^ General Surgery The Fifth Affiliated Hospital of Guangzhou Medical University Guangzhou P. R. China; ^3^ Key Laboratory of Biological Targeting Diagnosis, Therapy and Rehabilitation of Guangdong Higher Education Institutes Guangzhou P. R. China; ^4^ Logistics Department The First Affiliated Hospital of Sun Yat‐sen University Guangzhou P. R. China; ^5^ Division of Neonatology, Department of Pediatrics The Fifth Affiliated Hospital of Guangzhou Medical University Guangzhou P. R. China; ^6^ Department of Pathology University of California San Diego Health System San Diego California USA; ^7^ Jiangmen Key Laboratory of Clinical Biobank and Translational Research, Clinical Experimental Center Jiangmen Central Hospital Jiangmen China; ^8^ Department of Hepatobiliary, Pancreatic and Splenic Surgery, Jiangmen Central Hospital Affiliated Jiangmen Hospital of Sun Yat‐Sen University Jiangmen China; ^9^ Department of Pathology University of California Irvine Medical Center Orange California USA

**Keywords:** AKT1 signaling pathway, cell proliferation and cell cycle, hepatocellular carcinoma, reactive oxygen species, STOX1 isoform A

## Abstract

**Background:**

Dysregulation of transcription factors is one of the most common factors for the pathogenesis of hepatocellular carcinoma (HCC). To the best of our knowledge, no study has yet investigated the clinical significance and functional role of STOX1 in HCC.

**Methods:**

Real‐time PCR, Western blotting and immunohistochemistry were performed to examine the expression of STOX1‐A in HCC specimens. Animal experiment in vivo and functional cell assays in vitro were used to investigate the tumorigenic and proliferative ability of HCC cells. Luciferase and ROS assays were depolyed to investigate the molecular mechanisms underlying the biologic role of STOX1‐A in HCC.

**Results:**

In this study, we report that STOX1 isoform A (STOX1‐A) is significantly upregulated in HCC tissues, and elevated STOX1‐A levels are associated with poorer overall survival and progression‐free survival in HCC patients. Functional assays demonstrated that STOX1‐A upregulation promotes, whereas its silencing suppresses, HCC cell proliferation and growth both in vitro and in vivo. Mechanistic investigations revealed a dual mechanism by which STOX1‐A drives HCC progression. First, STOX1‐A transcriptionally upregulates cyclin B1, promoting cell proliferation. Second, it activates the AKT1 signaling pathway through reactive oxygen species (ROS)‐mediated deactivation of PTEN. Furthermore, a positive correlation between STOX1‐A expression and the levels of cyclin B1 and phosphorylated AKT1 (p‐AKT1 Ser473) was observed in clinical HCC samples.

**Conclusion:**

Our findings identify a novel dual mechanism by which STOX1‐A promotes HCC proliferation and growth, offering potential avenues for the development of anti‐tumor therapeutic strategies targeting STOX1‐A in HCC.

## 1 Introduction

Hepatocellular carcinoma (HCC) is one of the most prevalent cancers globally, with rising incidence and mortality rates [1]. It is the most common histological subtype of primary liver cancer, accounting for approximately 80% of all cases [2]. HCC also represents the fastest‐increasing cause of cancer‐related deaths, with an annual rise of 4.5% over recent decades. By 2025, the global incidence of HCC is projected to surpass 1 million cases [3, 4]. Consequently, identifying critical risk factors for HCC initiation and progression is essential for advancing prevention and treatment strategies.

Major risk factors for HCC include chronic hepatitis B virus (HBV) or hepatitis C virus (HCV) infection, heavy alcohol consumption, diabetes, exposure to aflatoxin‐contaminated food, and nonalcoholic fatty liver disease [4]. Genetic alterations, such as oncogene activation (e.g., K‐RAS) and tumor suppressor gene inactivation (e.g., TP53, PTEN), also play pivotal roles [5]. Furthermore, constitutive activation of key signaling pathways—including Wnt/β‐catenin, PI3K/AKT, JAK/STAT3, and NF‐κB—has been implicated in HCC progression [6]. Among these, the oncogenic role of AKT signaling has attracted substantial attention. This pathway is typically activated through cytokine‐receptor interactions involving EGF [7], IGF‐1 [8] and insulin [9]. Loss of PTEN function and mutations in components of the AKT pathway have been implicated in approximately 70% of aberrant AKT signaling in cancers [10, 11]. Notably, reactive oxygen species (ROS) also play a critical role in activating AKT signaling by oxidizing and inactivating PTEN [12, 13, 14], highlighting the importance of the ROS/PTEN/AKT axis in tumorigenesis.

Storkhead box 1 (STOX1), a transcription factor related to the FOX family, is predominantly expressed in brain tissue [15, 16]. It has been implicated in cell cycle progression and proliferation, localizing to centrosomes during mitosis [17, 18]. Among its six known isoforms, STOX1‐A and STOX1‐B are the most extensively studied [19]. Both isoforms share a putative DNA‐binding domain; however, only STOX1‐A possesses a transactivation domain, enabling it to regulate transcription [19]. Functional studies of STOX1‐A have primarily focused on non‐cancerous diseases such as Alzheimer's disease [20, 21, 22], preeclampsia [23, 24, 25, 26], and trophoblast dysfunction [27, 28]. Its role in cancer remains largely unexplored, with limited evidence suggesting its involvement in neuroblastoma [29] and glioma prognosis [30].

In this study, we demonstrate that STOX1‐A is significantly upregulated in HCC tissues, as confirmed by both our experimental findings and analyses of The Cancer Genome Atlas (TCGA) dataset. Elevated STOX1‐A expression is associated with poorer overall survival (OS) and progression‐free survival (PFS) in HCC patients. Functional assays revealed that STOX1‐A overexpression promotes, while its silencing suppresses, HCC cell proliferation and growth both in vitro and in vivo. Mechanistically, STOX1‐A drives HCC progression through two key pathways: transcriptional upregulation of cyclin B1 and activation of the AKT1 signaling cascade via ROS‐mediated PTEN inactivation. Moreover, STOX1‐A expression positively correlates with cyclin B1 and phosphorylated AKT1 (Ser473) levels in clinical HCC specimens. Collectively, these findings establish STOX1‐A as a clinically relevant and biologically significant factor in HCC and provide a foundation for the development of STOX1‐A–targeted therapeutic strategies.

## 2 Methods and Materials

### 2.1 Cell Lines and Cell Culture

HCC cell lines, including HepG2, Huh7, Hep 3B2.1‐7, Li‐7, and SK‐Hep‐1, were purchased from Procell Life Science & Technology Co. Ltd. (Wuhan, China). HepG2 (Cat. No. CL‐0103) was cultured in MEM medium supplemented with 10% fetal bovine serum (FBS) and 1% polystyrene (P/S); Huh7 (Cat. No. CL‐0120) cultured in DMEM medium supplemented with 10% FBS and 1% P/S; Hep 3B2.1‐7 (Cat. No. CL‐0102) cultured in MEM medium supplemented with 10% FBS and 1% P/S; Li‐7 (Cat. No. CL‐0139) cultured in RPMI‐1640 medium supplemented with 10% FBS and 1% P/S; and SK‐Hep‐1 (Cat. No. CL‐0212) cultured in MEM medium supplemented with 10% FBS and 1% P/S. SNU‐182 (Cat. No. H1‐2001) and SNU‐387 (Cat. No. H1‐7901) were purchased from Oricell Therapeutics (Shanghai, China) and cultured in RPMI‐1640 medium supplemented with 10% FBS and 1% P/S. All cell lines were grown under a humidified atmosphere of 5% CO_2_ at 37°C.

Primary hepatocytes (PHs) were obtained from the adjacent normal liver tissue. The detailed procedures were performed as follows: the normal liver tissue was placed in a 15 mL conical base centrifuge tube and washed with 1× phosphate‐buffered saline (PBS) three times; incubated the calvaria in 1% trypsin (1 mL) for 10 min at 37°C, removed and discarded the trypsin solution and washed in Supplemented Dulbecco's modified essential medium (sDMEM) two times (serum and calcium in the medium will inactivate any residual trypsin); incubated in 0.2% collagenase solution (800 μL) for 30 min at 37°C, and then removed the collagenase digest and discarded, and then replaced it with fresh collagenase solution for a further 60 min at 37°C; kept the final digest and transferred it to a 15 mL conical base centrifuge tube; washed tissues with sDMEM (5 mL) two times, transferred the solution to the 15 mL tube containing the final digest; spun the cell solution at 1500× g for 5 min at room temperature, discarded the supernatant and resuspended the cell pellet in sDMEM (1 mL); transferred the cell suspensions to 2 × 75 cm^2^ flask, and then add 20 mL of sDMEM 1 mL of cell suspension to the flask; incubate the flask under a humidified atmosphere of 5% CO_2_ at 37°C until the cells reached 80%–90% confluency (~3 days) for use.

### 2.2 Clinical Patients and Specimens

Patient consent and approval from the Institutional Research Ethics Committee from the Jiangmen Central Hospital were obtained prior to the use of these clinical materials for research purposes, and the approval number was [2023]91. A total of 10 paired matched adjacent normal tissues, 41 benign liver lesion tissues, and 131 paraffin‐embedded, archived HCC tissues were obtained during surgery or needle biopsy between January 2015 and May 2018, and were diagnosed based on clinical and pathological evidence. The clinicopathological features of the patients are summarized in Tables S2–S4.

### 2.3 Vectors and Retroviral Infection

Human STOX1 cDNA was purchased from (Vigene Biosciences, Shandong, China) and cloned into the pSin‐EF2 plasmid. Knockdown of endogenous STOX1 was performed by cloning two short hairpin RNA (shRNA) oligonucleotides into the pSUPER‐puro‐retro vector (OligoEngine, Seattle, WA, USA). The full sequence and two separate shRNA fragments of STOX1 are listed in Table S5. Small interfering RNA (siRNA) knockdown was obtained from Ribobio (Guangzhou, China). Transfection of siRNAs and plasmids was performed using Lipofectamine 3000 (Life Technologies) according to the manufacturer's instructions.

### 2.4 RNA Extraction, Reverse Transcription and Real‐Time PCR

Total RNA from cells was extracted using the Trizol reagent (Invitrogen) according to the manufacturer's instructions. The extracted RNA was pretreated with RNase‐free DNase, and 2 μg of RNA from each sample was used for cDNA synthesis primed with random hexamers. Complementary DNA (cDNA) was amplified and quantified using a CFX 96 Real‐time system (BIO‐RAD, USA) with iQ SYBR Green (Takara, Japan) according to the manufacturer's instructions. Expression levels of various genes were normalized to the housekeeping gene glyceraldehyde‐3‐phosphate dehydrogenase (GAPDH) as controls. The sequences of each primers are listed in Table S6. Gene expression data were analyzed using the comparative *Ct* method (2^−ΔΔ*Ct*^).

### 2.5 Western Blotting Analysis

Cells were lysed in radioimmunoprecipitation (RIPA) buffer (50 mM Tris, pH 7.4, 1% TX‐100, 0.2% Na deoxycholic acid, and 0.2% SDS, HALT complete tab [Roche]). Proteins were quantified using the Pierce BCA Protein Assay kit (Thermo Fisher Scientific). Equal amounts of protein were loaded per lane and resolved by SDS‐polyacrylamide electrophoresis. Proteins were transferred by semi‐dry electrophoresis (BioRad) onto PVDF (Millipore) and blocked by 5% nonfat milk for 1 h at room temperature. Membranes were incubated overnight at 4°C with anti‐cyclin D1 (1:1000, Cat. No. 55506S, Cell Signaling Technology), STOX1‐A (1:2000, Cat. No. ab189436, Abcam), cyclin E1 (1:1000, Cat. No. 20808S, Cell Signaling Technology), CDK1 (1:5000, Cat. No. ab133327, Abcam), cyclin A1 (1:5000, Cat. No. ab270940, Abcam), CDK2 (1:1000, Cat. No. 18048S, Cell Signaling Technology), cyclin B1 (1:1000, Cat. No. ab32053, Abcam), CDK4 (1:1000, Cat. No. 12790S, Cell Signaling Technology), CDK6 (1:1000, Cat. No. 13331S, Cell Signaling Technology), oxygenizing and reduction PTEN (1:1000, Cat. No. ab267787, Abcam), phosphorylated AKT1 (Ser473) (1:2000, Cat. No. 4060S, Cell Signaling Technology), total AKT1 (1:1000, Cat. No. 9559S, Cell Signaling Technology). Membranes were washed thrice (10 min each) in TBS‐T buffer and incubated for 40 min at room temperature with horseradish peroxidase‐conjugated anti‐mouse or anti‐rabbit secondary antibodies (GE Healthcare). Membranes were washed thrice (10 min each) in TBS‐T and developed on film with the ECL system. The membranes were stripped and reprobed with an anti–α‐tubulin antibody (1:1000, Cat. No. 9272S, Cell Signaling Technology) as the loading control. Nuclear/cytoplasmic fractionation was separated using the Cell Fractionation Kit (Cell Signaling Technology, USA) according to the manufacturer's instructions.

### 2.6 Anchorage‐Independent Growth Assay

A total of 500 cells were trypsinized and suspended in 2 mL of complete medium containing 0.3% agar (Sigma). This experiment was performed as previously described [31] and carried out three times independently for each cell line.

### 2.7 Cell Counting Kit‐8 Analysis

For cell counting kit‐8 analysis, cells (2 × 10^3^) were seeded into 96 well plates, and the specific staining process and methods were performed according to the previous study [32].

### 2.8 Colony Formation Assay

The cells were trypsinized as single cells and suspended in the media with 10% FBS. The indicated cells (300 cells per well) were seeded into a 6‐well plate for ~10–14 days. Colonies were stained with 1% crystal violet for 10 min after fixation with 10% formaldehyde for 5 min. Plating efficiency was calculated as previously described [33]. Different colony morphologies were captured under a light microscope (Olympus).

### 2.9 Cycle Cell Analysis

Cycle cell analysis was performed using the Cell Cycle and Apoptosis Analysis Kit (Cat No: C1052, Beyotime Biotechnology, Shanghai, China). Briefly, the cells in each well of a 6‐well plate (300 cells per well) were trypsinized as a single cell and suspended in the tube with PBS. Add 0.5 mL of propidium iodide (PI) staining solution to each tube of cell samples. Gently and thoroughly resuspend the cell pellet and incubate at 37°C in the dark for 30 min. After staining, samples can be stored protected from light at 4°C or on ice. Flow cytometry is performed within 24 h of staining. Use a flow cytometer to detect red fluorescence at an excitation wavelength of 488 nm, while also measuring light scattering. Use appropriate analysis software to evaluate cellular DNA content and light scattering.

### 2.10 ROS Assay

ROS assay was performed using the Reactive Oxygen Species Assay Kit, also known as the ROS Assay Kit (Cat No: S0033S, Beyotime Biotechnology, Shanghai, China). Dilute DCFH‐DA at a ratio of 1:1000 using Extracellular Solution (C0216), or alternatively with PBS, HBSS, or other suitable solutions, to a final concentration of 10 μmol/L. After collecting the cells, resuspend them in the diluted DCFH‐DA at a concentration of 1–20 million cells/mL. Incubate in a 37°C incubator for 20 min, gently mixing every 3–5 min to ensure sufficient contact between the probe and cells. Wash the cells three times using Extracellular Solution (C0216), or alternatively with PBS, HBSS, serum‐free medium, or other suitable solutions, to thoroughly remove any DCFH‐DA that has not entered the cells. Cells can then be stimulated directly with a ROS‐positive control or the drug of interest, or divided into several portions for stimulation. Typically, ROS levels can be significantly increased 20–30 min after stimulation with a positive control. Add Rosup only to the positive control wells as the ROS‐positive control; there is no need to add Rosup to other wells. It is recommended to use Extracellular Solution (C0216), PBS (C0221A), and Hanks' Balanced Salt Solution (HBSS, C0218). Beyotime also offers a ROS detection kit (S0034) that includes optimized DCFH‐DA dilution solution and Rosup dilution solution for easier use and more stable, reliable detection results. For samples loaded with the probe in situ, you can directly observe them using a laser confocal microscope or collect the cells for detection using a fluorescence spectrophotometer, fluorescence microplate reader, or flow cytometer. For samples in which the probe was loaded after cell collection, detection can be performed using a fluorescence spectrophotometer, fluorescence microplate reader, or flow cytometer. Direct observation under a laser confocal microscope is also possible. Use an excitation wavelength of 488 nm and an emission wavelength of 525 nm to detect changes in fluorescence intensity in real time or at different time points before and after stimulation. The fluorescence spectrum of DCF is very similar to that of FITC, so DCF can be detected using FITC settings.

### 2.11 Immunohistochemistry

The immunohistochemical (IHC) procedure refers to the previously described study [34]. Briefly, paraffin‐embedded specimens were serially cut into 4 μm sections and baked at 65°C for 30 min. The sections were deparaffinized with xylenes and rehydrated. Sections were submerged into EDTA antigenic retrieval buffer and microwaved for antigenic retrieval. The sections were treated with 3% hydrogen peroxide in methanol to quench the endogenous peroxidase activity, followed by incubation with 1% bovine serum albumin to block nonspecific binding. Anti‐STOX1‐A (1:2000, Cat. No. PA5‐98549, Invitrogen), cyclin B1 (1:1000, Cat. No. 61976S, Cell Signaling Technology), phosphorylated AKT1 (Ser473) (1:2000, Cat. No. 4060S, Cell Signaling Technology) and Ki‐67 (1:400, Cat. No. 9027S, Cell Signaling Technology) were incubated with the sections overnight at 4°C. After washing, the tissue sections were treated with biotinylated anti‐rabbit or anti‐mouse secondary antibody (Zymed, San Francisco, CA), followed by further incubation with streptavidin‐horseradish peroxidase complex (Zymed). Staining of tissue sections was performed using 3,3′‐diaminobenzidine (DAB). Sections were counterstained with hematoxylin, followed by dehydration and mounting. The degree of immunostaining of formalin‐fixed, paraffin‐embedded sections was reviewed and scored independently by two independent investigators, based on both the proportion of positively stained tumor cells and the intensity of staining. The proportion of tumor cells was scored as follows: 0 (no positive tumor cells); 1 (< 10% positive tumor cells); 2 (10%–35% positive tumor cells); 3 (35%–70% positive tumor cells) and 4 (> 70% positive tumor cells). The staining intensity was graded according to the following criteria: 0 (no staining); 1 (weak staining, light yellow); 2 (moderate staining, yellow brown) and 3 (strong staining, brown). The staining index (SI) was calculated as staining intensity score × proportion of positive tumor cells. Using this method of assessment, we evaluated the expression of STOX1A, Cyclin B1, p‐AKT (Ser473) and Ki‐67 in tissues by determining SI, with scores of 0, 1, 2, 3, 4, 6, 8, 9, or 12. 16 representative staining fields of each section were analyzed to procure the staining index score. Protein expression was further evaluated based on the high and low level expression in HCC samples (cutoff is defined as the staining index of 4).

### 2.12 Animal Study

Eight‐week‐old BALB/c‐nu mice were obtained from the Guangdong Medical University Experimental Animal Center. Mice should be housed in pathogen‐free conditions with a 12‐h light/dark cycle. Temperature and humidity are maintained at 20°C–24°C with 40%–60% humidity. Individually ventilated cages with appropriate bedding material are used for each mouse, and standard chow and autoclaved water are provided. Daily checks are performed for signs of distress, infection, or suffering of the mice. Six mice were randomly designated to each group. Hep G2 cells (1 × 10^6^) were injected into the subcutaneous tissue of the inguinal fold in each mouse with a 300 μL 28 g ½ insulin syringe. Mice were monitored every 3 days to measure the size of the tumor in the subcutaneous tissue of the inguinal fold and log the size accordingly until week 5. The mice were euthanized at the designated endpoints, and the tumor tissues in each mouse were further sectioned and evaluated. Tumor sections were subjected to hematoxylin and eosin (H&E) staining, as well as IHC staining for STOX1 and Ki‐67 to assess histological features. Tumor cell numbers were quantified as previously described [35].

### 2.13 Statistical Analysis

Statistical analyses were performed using GraphPad 5.0 software (USA). Differences between two groups were evaluated using Student's *t*‐test, while one‐way ANOVA was used for comparisons among multiple groups. A *p* < 0.05 was considered statistically significant. All experiments were performed in triplicate.

## 3 Results

### 3.1 STOX1‐A Is Upregulated in HCC

**FIGURE 1:**
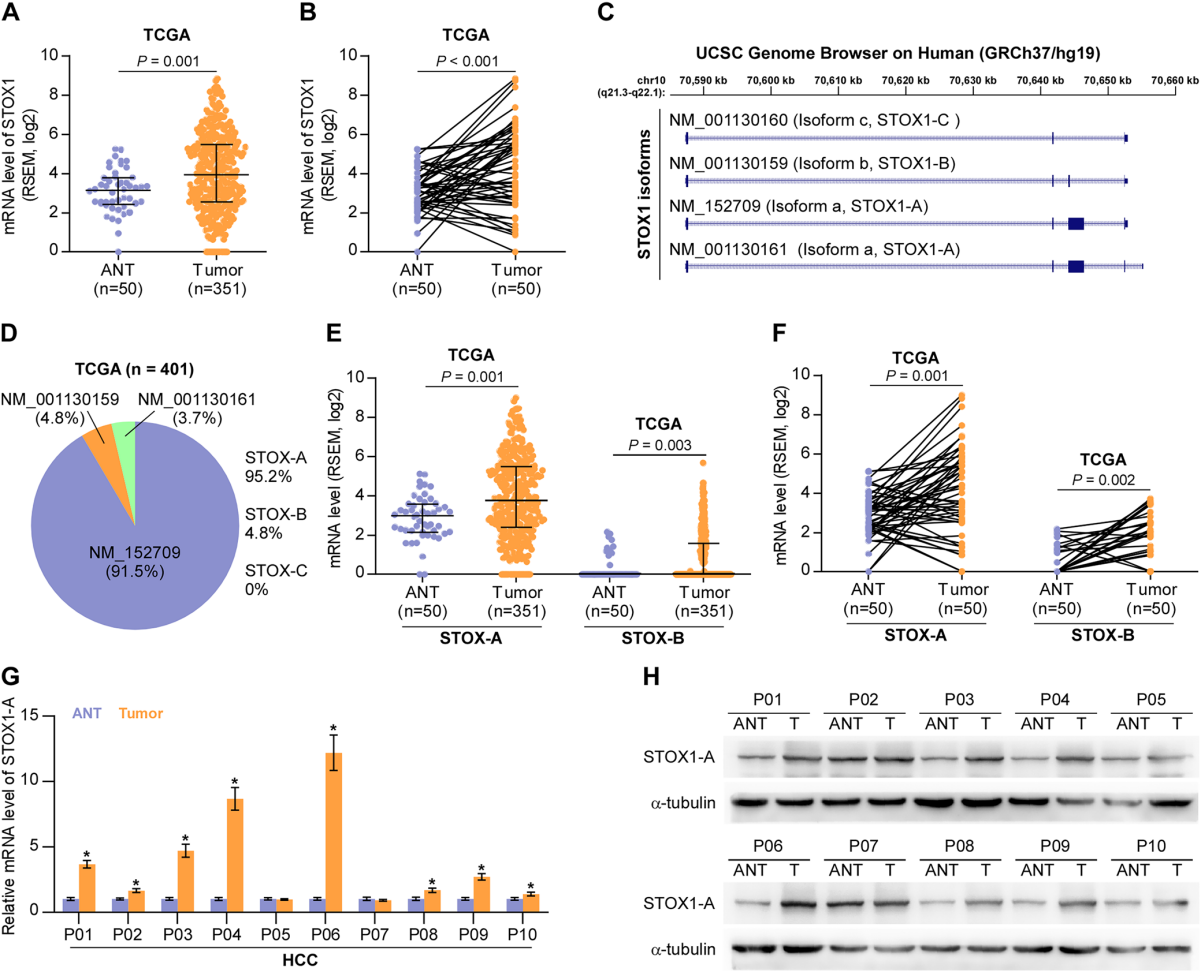
STOX1‐A is upregulated in HCC. (A) STOX1 expression in 50 adjacent normal tissues (ANT) and 351 HCC tissues in HCC dataset from TCGA. (B) STOX1 expression in 50 paired ANT and HCC tissues in HCC dataset from TCGA. (C) Analysis of STOX1 isoform using UCSC genome. (D) Different isoform expression of STOX1, including STOX1‐A STOX1‐B, and STOX1‐C, depicted in bar graph in HCC dataset from TCGA. (E) STOX1‐A and STOX1‐B expression in 50 adjacent normal tissues (ANT) and 351 HCC tissues in HCC dataset from TCGA. (F) STOX1‐A and STOX1‐B expression in 50 paired ANT and HCC tissues in HCC dataset from TCGA. (G, H) Real‐time PCR (G) and Western blot (H) analysis of STOX1‐A expression in 10 clinical paired ANT and HCC tissues.

First, we performed an in silico analysis of STOX1 expression using the publicly available TCGA HCC dataset. This analysis revealed that STOX1 expression was significantly elevated in HCC tissues compared to adjacent nontumor tissues (ANT) (Figure 1A), as well as in matched paired ANT samples (Figure 1B). Genome analysis from the University of California Santa Cruz (UCSC) identified three transcript isoforms of STOX1: STOX1‐A, STOX1‐B, and STOX1‐C (Figure 1C). Expression levels of these isoforms were further examined in the TCGA dataset. The results showed that STOX1‐A was the predominant isoform, accounting for approximately 91.5% of total STOX1 expression, followed by STOX1‐B at 4.8% and STOX1‐C at 3.7% (Figure 1D). Notably, the differential expression of STOX1‐A between HCC and ANT tissues was more pronounced than that of STOX1‐B (Figure 1E,F). Based on these findings, STOX1‐A was selected for further investigation in the context of HCC. Consistent with the TCGA data, STOX1‐A expression was also found to be upregulated in clinical HCC tissue samples (Figure 1G,H). These results suggest that STOX1‐A upregulation may play a role in the development and progression of HCC.

### 3.2 High Expression of STOX1‐A Correlates With Worst Prognosis

**FIGURE 2:**
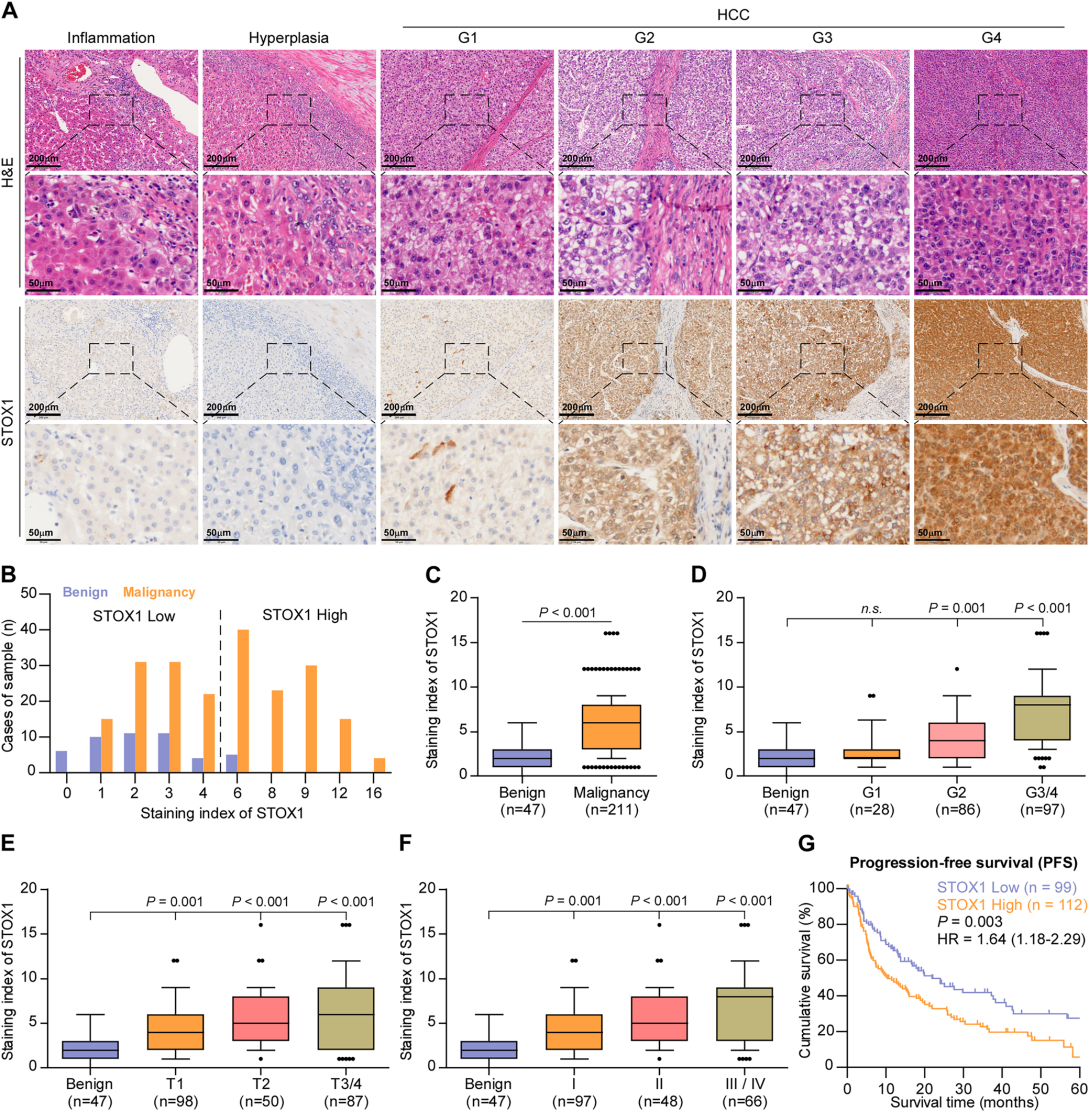
High expression of STOX1‐A correlates with poor prognosis in HCC patients. (A) Analysis of STOX1 expression in benign inflammatory liver lesion, benign hyperplastic liver lesion and HCC with different grades using immunohistochemical (IHC) staining. Upper panel: 4× magnification, scale bar, 200 μm; lower panel: 20× magnification, scale bar, 50 μm. (B) The number of different staining index of STOX1‐A in benign and HCC tissues. (C) Staining index of STOX1‐A in benign and malignant HCC tissues. (D) Staining index of STOX1‐A in benign and HCC tissues with different grade. n.s., no significant difference. (E) Staining index of STOX1‐A in benign and HCC tissues with different T stage. (F) Staining index of STOX1‐A in benign and HCC tissues with different clinical stage. (G) Kaplan–Meier progression‐free survival analysis of HCC patients stratified by high and low STOX1‐A levels.

Forty‐one benign liver lesion specimens and 131 HCC specimens were used to further evaluate the clinical significance of STOX1‐A in HCC patients by IHC. The representative sections of inflammatory liver disease, liver parenchyma with hyperplasia, and HCC with different grades from I to IV respectively are shown in Figure 2A. As a transcription factor, it is conceivable that nuclear STOX1‐A expression was expected (Figure 2A), and STOX1‐A upregulation was observed in HCC compared to the benign liver lesions (Figure 2B,C). Furthermore, STOX1‐A expression was further increased with the advancement of tumor grade (Figure 2D, Table S1), T stage (Figure 2E) and clinical stage (Figure 2F). HCC patients with high STOX1‐A levels showed poorer PFS compared with those with low STOX1‐A levels (Figure 2G). The clinical correlation of STOX1‐A expression was further explored in the HCC dataset from TCGA. Consistently, STOX1‐A expression gradually increased with the advancement of tumor grade (Figure S1A), T stage (Figure S1B) and clinical stage (Figure S1C), and the overexpression of STOX1‐A was associated with shorter overall and PFS (Figure S1D,E). Thus, our data imply that a high level of STOX1‐A is positively correlated with the worst prognosis in HCC patients.

### 3.3 STOX1‐A Promotes HCC Cells Proliferation and Growth

**FIGURE 3:**
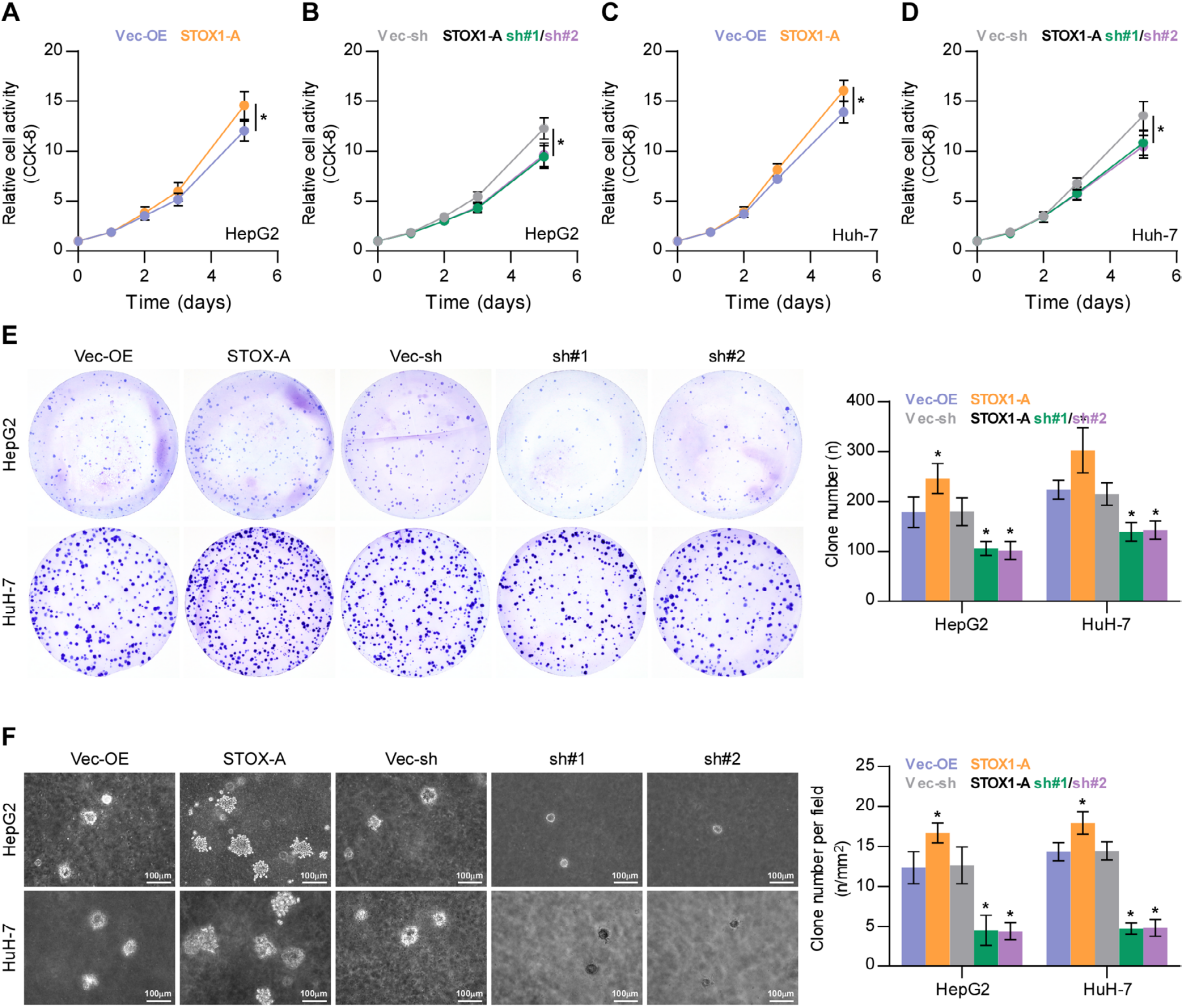
STOX1‐A promotes proliferation of HCC cells in vitro. (A–D) The effect of STOX1‐A overexpression or downexpression on proliferation of HCC cells using CCK‐8 assay. *p < 0.05. (E) The effect of STOX1‐A overexpression or downexpressio on proliferation of HCC cells using colony‐formation assay. *p < 0.05. (F) The effect of STOX1‐A overexpression or downexpressio on proliferation of HCC cells using soft agar colony‐formation assay. *p < 0.05.

To determine the functional role of STOX1‐A in HCC, the expression levels of STOX1‐A were first examined in HCC cell lines. STOX1‐A expression was differentially increased in 5 out of 7 HCC cell lines, including HepG2, Huh7, Li‐7, SNU‐387, and SK‐Hep‐1, compared to the PHs (Figure S2A,B). The STOX1‐A‐stably overexpressing and downexpressing cell lines were constructed in HepG2 and Huh7 HCC cells, respectively (Figure S2C,D). Upregulating STOX1‐A increased, while silencing STOX1‐A reduced the HCC cells proliferation and growth (Figure 3A–E). The soft‐agar colony‐formation assay revealed that upregulating STOX1‐A increased, while silencing STOX1‐A reduced HCC cells growth on the soft‐agar (Figure 3F). These findings demonstrated that overexpression of STOX1‐A promotes HCC cells proliferation and growth in vitro.

**FIGURE 4:**
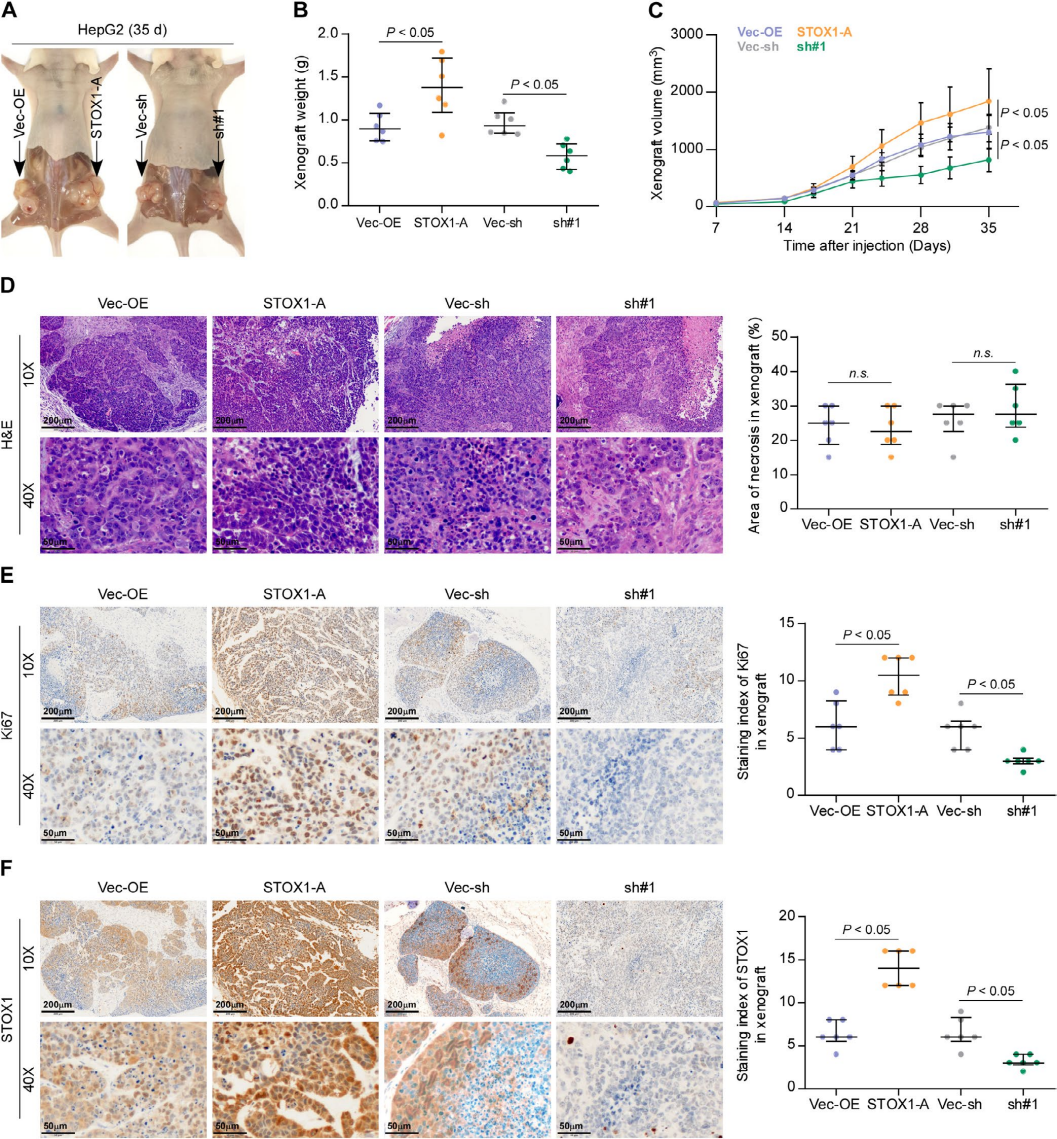
STOX1‐A promotes tumorigenesis of HCC cells in vivo. (A) Schematic model of subcutaneous injection of the indicated tumor cells in vivo. (B, C) The effect of STOX1‐A overexpression or downexpression on the tumor weight (B) and volume (C) in the indicated mice group, including vector‐overexpression group (Vec‐OE), STOX1‐A overexpression group (STOX1A), vector‐shRNA group (Vec‐sh) and STOX1‐A overexpression group (sh#1). (D) H&E staining analysis of necrotic areas in the tumors from the indicated mice group (left panel). Analysis of necrotic areas in the indicated tumor tissues (right panel). n.s., no significant difference. (E, F) Analysis of Ki‐67 (E) and STOX1‐A (F) expression in the tumor tissues from the indicated mice group.

Then, the effect of STOX1‐A was further investigated in vivo, where the vector‐overexpression (Vec‐OE), STOX1‐A‐overexpressing (STOX1‐A), vector‐shRNA (Vec‐sh) and STOX1‐A—silencing (sh#1) Hep G2 cells were used. We found that upregulating STOX1‐A enhanced the tumor weight and volume, as well as the Ki‐67 staining index compared with the Vec‐OE group (Figure 4B–E), but had no effect on necrotic formation in tumor tissues (Figure 4D). Conversely, silencing STOX1‐A significantly reduced the tumor weight, volume, and Ki‐67 staining index compared with the Vec‐sh group (Figure 4B–E). Additionally, STOX1‐A expression was significantly upregulated in the STOX1‐A group of mice compared with that in Vec‐OE, and decreased in the sh#1 group compared with the Con‐sh group of mice (Figure 4F). Taken together, these findings demonstrated that STOX1‐A promotes HCC cell proliferation and growth in vivo and in vitro.

### 3.4 STOX1‐A Promotes HCC Cells Proliferation and Growth by Transcriptionally Upregulating CCNB1

**FIGURE 5:**
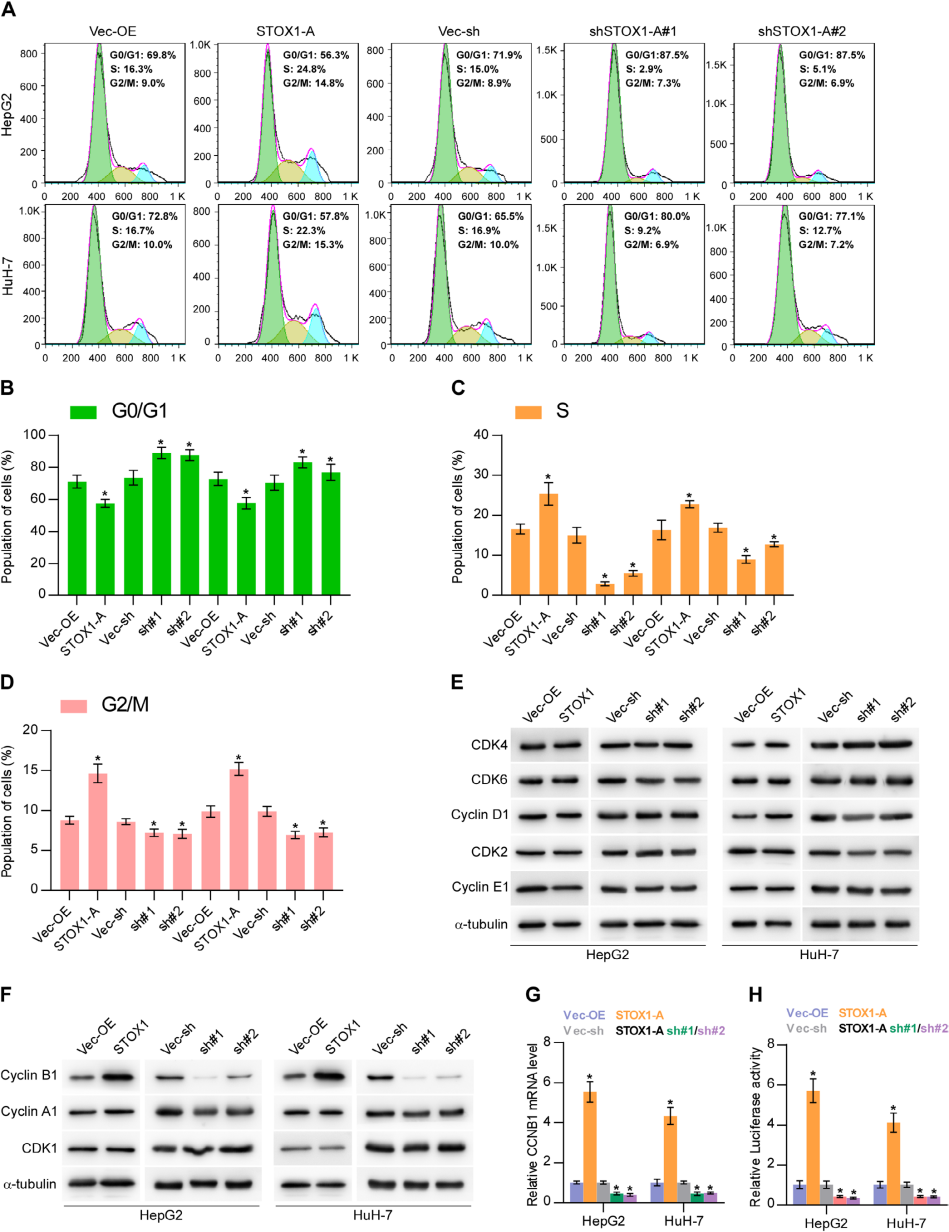
STOX1‐A promotes proliferation and growth of HCC cells by transcriptionally upregulating CCNB1. (A–D) The effect of STOX1‐A overexpression or downexpression on cell cycle progression of HCC cells, including G0/G1 phases (B), S phase (C) and G2/M phases (D), using flow cytometry. (E, F) Western blot analysis of the effect of STOX1‐A overexpression or downexpression on the expression of G1/S‐related regulatory proteins, including CDK2, CDK4, CDK6, cyclin D1 and cyclin E1, and G2/M‐related regulatory protein, including cyclin A1, cyclin B1 and CDK1. (G) Real‐time PCR analysis of the effect of STOX1‐A overexpression or downexpression on cyclin B1 expression. (H) Analysis of the effect of STOX1‐A overexpression or downexpression on the luciferase activity of cyclin B1 promoter using luciferase activity assay. * indicates *p* < 0.05.

As well known, cell cycle progression has been extensively demonstrated to be a critical factor in cell proliferation and growth [36, 37, 38]. Therefore, the role of STOX1‐A in cell cycle regulation was first examined. As shown in Figure 5A–D, upregulating STOX1‐A significantly increased the percentage of S and G2/M phases but decreased the percentage of G0/G1 phases; in contrast, silencing STOX1‐A enhanced the percentage of G0/G1 phases but reduced the percentage of S and G2/M phases. Then, we further examined the influence of STOX1‐A on cell cycle‐related proteins and found that neither upregulating nor downregulating STOX1‐A had an influence on the expression of G1/S‐related regulatory proteins (Figure 5E). However, upregulating STOX1‐A increased, while silencing STOX1‐A reduced the expression levels of the G2/M‐related regulatory protein, cyclin B1, but had no or mild effect on other G2/M‐related regulatory proteins, including cyclin A1 and CDK1 (Figure 5F). Moreover, upregulating STOX1‐A increased, while silencing STOX1‐A decreased the mRNA levels of cyclin B1 in HCC cells (Figure 5G). Luciferase assay further showed that STOX1‐A upregulation elevated, while STOX1‐A downregulation reduced the luciferase activity of the promoter of cyclin B1 (Figure 5H). Our findings suggest that STOX1‐A promotes HCC cell proliferation and growth by promoting the cell cycle, which is largely dependent on transcriptional upregulation of CCNB1 by STOX1‐A.

### 3.5 STOX1‐A Promotes Proliferation and Growth by ROS/PTEN/AKT1 Pathway

**FIGURE 6:**
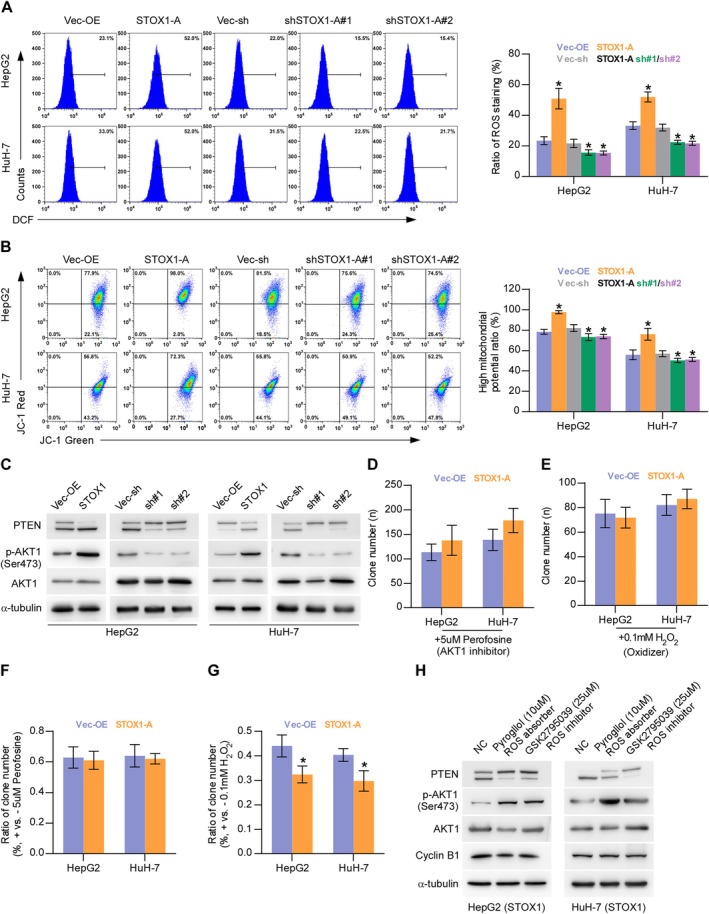
STOX1‐A promotes proliferation and growth by ROS/PTEN/AKT1 pathway. (A) The effect of STOX1‐A overexpression or downexpression on intracellular production of ROS in HCC cells. *p < 0.05. (B) The effect of STOX1‐A overexpression or downexpression on mitochondrial membrane potential using mitochondrial membrane potential assay. *p < 0.05. (C) Western blot analysis of the effect of STOX1‐A overexpression or downexpression on the expression of oxygenizing and reduction PTEN, phosphorylated AKT1 (Ser473) and total AKT1. (D, E) The effect of AKT1 signaling inhibitor, Perofosine, (5 μmol/L) or H_2_O_2_ (100 μmol/L) on colony‐formation ability in the indicated HCC cells using colony‐formation assay. (F, G) The effect of Perofosine (5 μmol/L) (F) or H_2_O_2_ (100 μmol/L) (G) on colony‐formation ratio in the indicated HCC cells compared to the original levels using colony‐formation assay. *p < 0.05. (H) Western blot analysis of the effect of oxygen species absorber‐Pyrogallol or inhibitor‐GSK2795039 on the expression of oxygenizing PTEN, total PTEN, phosphorylated AKT1 (Ser473) and total AKT1 in the indicated HCC cells.

Notably, overexpression of STOX1‐A upregulated ROS [23, 39], which further activated the AKT signaling pathway by oxygenizing PTEN, leading to the deactivation of PTEN [12, 13, 14]. The AKT signaling pathway has been well‐documented to be critical in cell proliferation and growth [40, 41, 42]. Therefore, we first examined the influence of STOX1‐A on ROS. Upregulating STOX1‐A enhanced, while STOX1‐A downregulated the intracellular levels of ROS in HCC cells. Additionally, STOX1‐A upregulation increased, while STOX1‐A downregulation decreased the membrane potential of mitochondria (Figure 6B). STOX1‐A upregulation increased, while STOX1‐A downregulation decreased the oxygenized PTEN and phosphorylated AKT1 at Ser473 (p‐AKT1 (Ser473)) in HCC cells and reduced the reduction of PTEN but had no significant influence on total AKT1 expression (Figure 6C). Interestingly, the addition of the AKT1 signaling inhibitor, Perofosine, or H_2_O_2_, differentially suppressed the colony‐formation abilities of control and STOX1‐A overexpression HCC cells (Figure 6D,E). Notably, Perofosine reduced both the colony‐formation ratio of control and STOX1‐A overexpression HCC cells to 60% compared to original levels (Figure 6F). However, the suppressive effect of H_2_O_2_ on the colony‐formation ratio in STOX1‐A overexpressing HCC cells was more robust compared with the Vec‐OE (Figure 6G), which may be explained by the evidence that upregulating STOX1‐A increased, while silencing STOX1‐A decreased the intracellular levels of ROS in HCC cells (Figure 6A). Notably, either the oxygen species absorber Pyrogallol or the inhibitor GSK2795039 significantly attenuated the stimulatory effect of STOX1‐A overexpression on the expression levels of the oxygenized PTEN and p‐AKT1 (Ser473) in HCC cells, but had no significant influence on total PTEN and AKT1 expression (Figure 6H), as well as the growth of STOX1‐A overexpressing HCC cells (Figure S3A). Taken together, STOX1‐A promotes HCC cell proliferation and growth by the ROS/PTEN/AKT1 pathway.

### 3.6 Clinical Correlation of STOX1‐A Expression With Cyclin B1 and p‐AKT1 (Ser473) in Clinical HCC Samples

**FIGURE 7:**
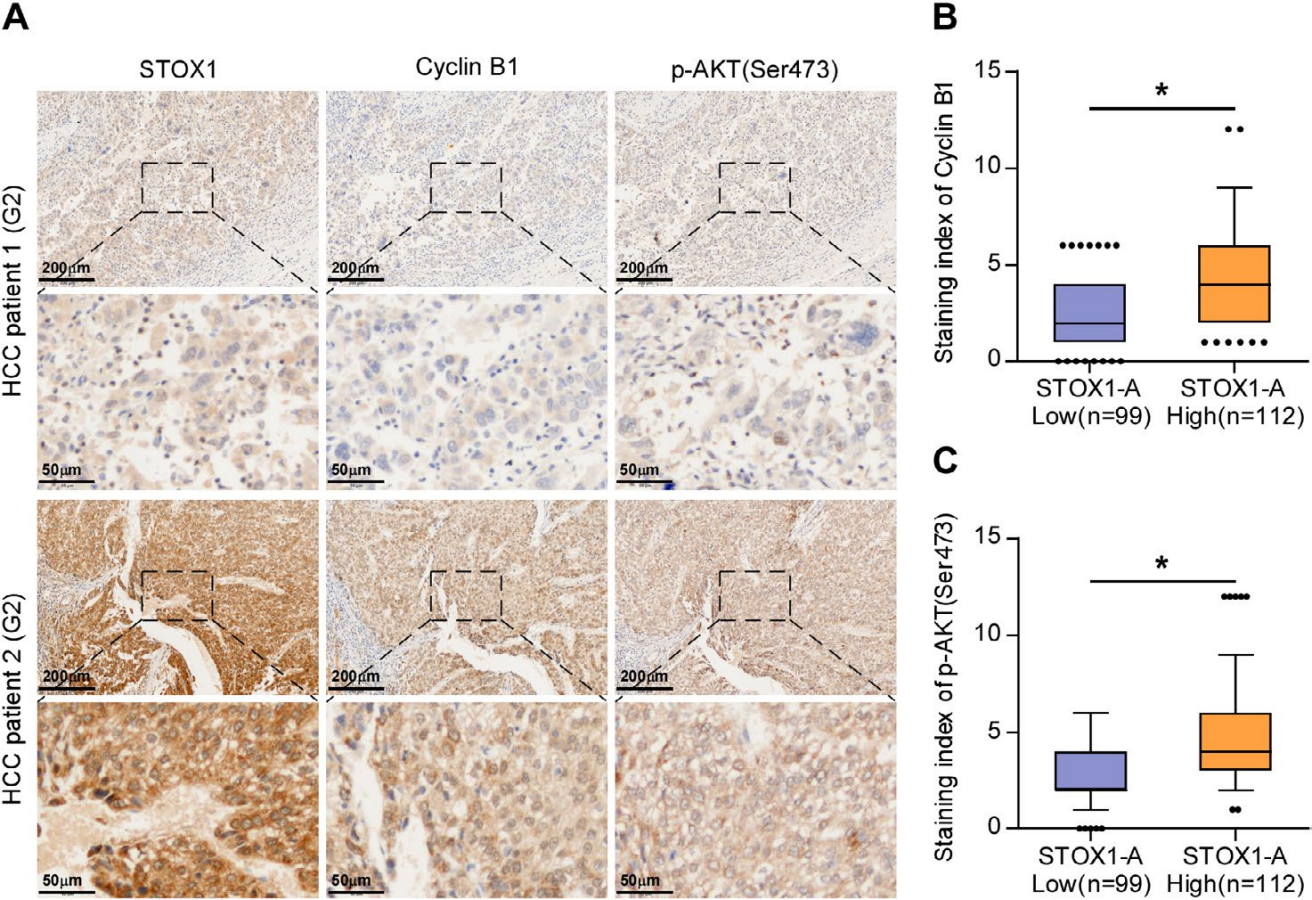
Clinical correlation of STOX1‐A expression with cyclin B1 and p‐AKT (Ser473) in clinical HCC samples. (A) Analysis of clinical correlation of STOX1‐A expression with cyclin B1 and p‐AKT (Ser473) in clinical HCC samples using immunohistochemical (IHC) staining. Upper panel: 4× magnification, scale bar, 200 μm; lower panel: 20× magnification, scale bar, 50 μm. (B, C) Statistical analysis of STOX1‐A expression with cyclin B1 (B) and p‐AKT (Ser473) (C) expression in clinical HCC samples. *p < 0.05.

Finally, the clinical correlation of STOX1‐A expression with cyclin B1 and p‐AKT1 (Ser473) in clinical HCC samples was further investigated. By IHC staining, we found that STOX1‐A, cyclin B1, and p‐AKT1 (Ser473) were all predominantly expressed in the nucleus of tumor cells (Figure 7A). Statistical analysis further revealed that STOX1‐A expression was positively and significantly correlated with cyclin B1 and p‐AKT1 (Ser473) expression in clinical HCC specimens (Figure 7B,C). These findings clinically support the notion that STOX1‐A promotes HCC cell proliferation and growth dependent on both the activity of cyclin B1 and the AKT1 pathway.

## 4 Discussion

Previous studies on STOX1 have primarily focused on its involvement in non‐cancerous conditions such as Alzheimer's disease [20, 21, 22], preeclampsia [23, 24, 25, 26], and trophoblast dysfunction [27, 28]. In the context of cancer, limited research has reported STOX1 overexpression in colon adenocarcinoma [43] and its downregulation in medulloblastoma [44] and glioma [30]. However, to our knowledge, no studies have investigated the specific isoforms of STOX1, nor their clinical relevance or functional roles in cancer. In this study, we identified STOX1‐A as the predominant isoform upregulated in HCC tissues. High STOX1‐A expression was significantly associated with poorer OS and PFS, suggesting its clinical importance in HCC. Functional gain‐ and loss‐of‐function assays demonstrated that STOX1‐A promotes cell cycle progression, highlighting its oncogenic role as a key regulator of cell cycle dynamics in HCC.

Since its discovery, STOX1‐A has been shown to function predominantly as a transcriptional activator in several diseases, including neurodegeneration [16], preeclampsia [25], and neuroblastoma [29]. Interestingly, in certain contexts, STOX1‐A has also been reported to act as a transcriptional repressor, contributing to Alzheimer's disease [21] and trophoblast dysfunction [24, 27]. These findings suggest that the transcriptional activity of STOX1‐A is highly context‐dependent. In the present study, our data support a transcriptional activating role for STOX1‐A in HCC. Specifically, STOX1‐A overexpression increased both the mRNA expression and promoter luciferase activity of CCNB1 (cyclin B1), indicating its function as a transcriptional activator in this cancer type. Together, these results underscore the oncogenic potential of STOX1‐A in HCC via transcriptional regulation of cell cycle–related genes.

CCNB1 is a key regulatory protein involved in the cell cycle, particularly during the G2/M transition [45]. Numerous studies have shown that aberrant expression of CCNB1 plays a critical role in accelerating cell cycle progression, thereby promoting uncontrolled cell proliferation and rapid tumor growth [46, 47], as well as contributing to therapeutic resistance [48]. Notably, multiple mechanisms—at both genetic and epigenetic levels—have been implicated in the dysregulation of CCNB1 expression, including transcriptional regulation, DNA methylation, and modulation by non‐coding RNAs such as microRNAs, all of which contribute to tumorigenesis and cancer progression across various malignancies [49, 50, 51]. In this study, our findings demonstrate that STOX1‐A markedly enhanced cell cycle progression and proliferation of HCC cells. Specifically, STOX1‐A overexpression increased the proportion of cells in the S and G2/M phases while reducing the proportion in the G0/G1 phases. This was accompanied by elevated mRNA and protein levels of CCNB1. Mechanistically, luciferase reporter assays confirmed that STOX1‐A transcriptionally upregulates CCNB1 expression. These results suggest that STOX1‐A promotes HCC cell proliferation and growth through transcriptional activation of CCNB1.

The dysregulation of the ROS/PTEN/AKT signaling axis has been widely implicated in the development and progression of various cancers and other diseases. Lu et al. reported that hydrogen alleviated peritoneal fibrosis by reducing intracellular ROS levels, thereby inhibiting the PTEN/AKT pathway [52]. Indeed, ROS overproduction can inactivate PTEN by promoting its oxidation [14], and PTEN inactivation is extensively reported to be significantly related to constitutive AKT signaling activation [10, 11]. Additionally, ROS has been shown to directly activate AKT signaling [12, 13]. Conversely, several independent studies have indicated that ROS can repress AKT signaling by increasing PTEN expression, thereby inhibiting the proliferation of cardiac muscle cells [53, 54]. Interestingly, ROS has been reported to exert both activating and inhibitory effects on PTEN and AKT signaling in prostate cancer DU‐145 cells and acute myeloid leukemia cells [55, 56]. These findings highlight the complexity of the crosstalk between ROS and PTEN/AKT signaling, which appears to be highly context‐dependent. In this study, we found that STOX1‐A upregulation increased, while its silencing reduced intracellular ROS levels in HCC cells. Notably, STOX1‐A overexpression enhanced, whereas silencing STOX1‐A abrogated the inhibitory effect of hydrogen peroxide (H_2_O_2_) on HCC cell colony‐forming ability. Further analyses revealed that ROS activated AKT1 signaling by oxidizing and inactivating PTEN, thereby promoting cell cycle progression and proliferation in HCC cells. These findings uncover a novel mechanism whereby STOX1‐A drives HCC progression through ROS‐mediated PTEN inactivation and subsequent AKT1 activation.

Several transcription factors, including p53 [57, 58, 59], HIF‐1α [60, 61, 62], BRCA1/2 [63, 64, 65] have been investigated as potential biomarkers for cancer detection, diagnosis, and prognosis. In HCC, m6A regulators such as HNRNPC have emerged as key prognostic indicators and potential therapeutic targets, especially in the context of immunotherapy, underscoring the importance of m6A‐regulated patterns in stratifying HCC patients for personalized treatment [66]. Notably, STOX1 downregulation has been associated with advanced WHO grades and poorer outcomes in glioma, suggesting its potential as a prognostic biomarker [30]. In our study, STOX1‐A was significantly upregulated in HCC tissues. Clinical analyses using both our specimens and data from TCGA revealed that high STOX1‐A expression correlated with worse OS and PFS in HCC patients. These findings support STOX1‐A as a promising prognostic biomarker in HCC, although further validation in larger, prospective cohorts is warranted.

In summary, this study demonstrates that STOX1‐A promotes HCC cell proliferation and growth through transcriptional upregulation of CCNB1 and ROS‐mediated activation of AKT1 signaling. A deeper understanding of the STOX1‐A/ROS/PTEN/AKT1 axis provides a solid theoretical foundation for developing STOX1‐A–targeted therapies in HCC.

## Author Contributions

**Chunlin Jiang:** conceptualization (equal), supervision (lead), writing – review and editing (equal). **Chong Wang:** data curation (equal), formal analysis (equal). **Jian Ao:** data curation (equal), formal analysis (equal). **Yangping Liu:** writing – original draft (supporting). **Fengjie Sun:** data curation (supporting). **Wangpan Shi:** data curation (supporting), writing – original draft (supporting). **Zeyi Guo:** formal analysis (supporting). **Yanping Wu:** formal analysis (supporting). **Luxiang Gan:** validation (supporting). **Meimei Wu:** data curation (supporting). **Yaofeng Zhi:** writing – original draft (supporting). **Zijie Meng:** data curation (supporting), formal analysis (supporting). **Wanting Wu:** data curation (supporting). **Juanhua Wu:** formal analysis (supporting). **Yong Ye:** methodology (supporting). **Xin Zhang:** conceptualization (equal), data curation (equal), formal analysis (equal), funding acquisition (equal), investigation (equal), methodology (equal), project administration (equal), supervision (equal), validation (equal), visualization (equal), writing – original draft (equal), writing – review and editing (equal). **Dong Ren:** conceptualization (lead), data curation (lead), formal analysis (lead), funding acquisition (lead), investigation (lead), methodology (lead), supervision (lead), validation (lead), visualization (lead), writing – original draft (lead), writing – review and editing (lead). **Mingxin Pan:** conceptualization (equal), data curation (equal), formal analysis (equal), funding acquisition (equal), investigation (equal), methodology (equal), project administration (equal), supervision (equal), validation (equal), visualization (equal), writing – original draft (equal), writing – review and editing (equal).

## Ethics Statement

Approved by the Institutional Animal Research Ethics Committee (GDY2302284).

## Consent

Obtained.

## Conflicts of Interest

The authors declare no conflicts of interest.

## Supporting information


Data S1.

## Data Availability

Data sharing not applicable to this article as no datasets were generated or analysed during the current study.
