# Supplementary material for: STOX1 Isoform A Promotes Proliferation and Progression of Hepatocellular Carcinoma by Dual Mechanisms of Transcriptionally Upregulation of Cyclin B1 and Activation of ROS‐Dependent PTEN/AKT1 Signaling
Source: Cancer Med. 2025 Jun 26;14(13):e70958. doi: 10.1002/cam4.70958 (PMC12198656; doi:10.1002/cam4.70958)
Supplement: Supplementary file 1 — Data S1. [file CAM4-14-e70958-s001.docx]

**Supplementary figure 1:**

(A) Expression levels of STOX1-A in benign and HCC tissues with different grade in HCC dataset from TCGA. n.s. indicates no significant difference. (B) Expression levels of STOX1-A in benign and HCC tissues with different T stage in HCC dataset from TCGA. (C) Expression levels of STOX1-A in benign and HCC tissues with different clinical stage in HCC dataset from TCGA. n.s. indicates no significant difference. (D and E) Kaplan-Meier progression-free survival (D) and overall survival (E) analysis of HCC patients stratified by high and low STOX1-A levels.


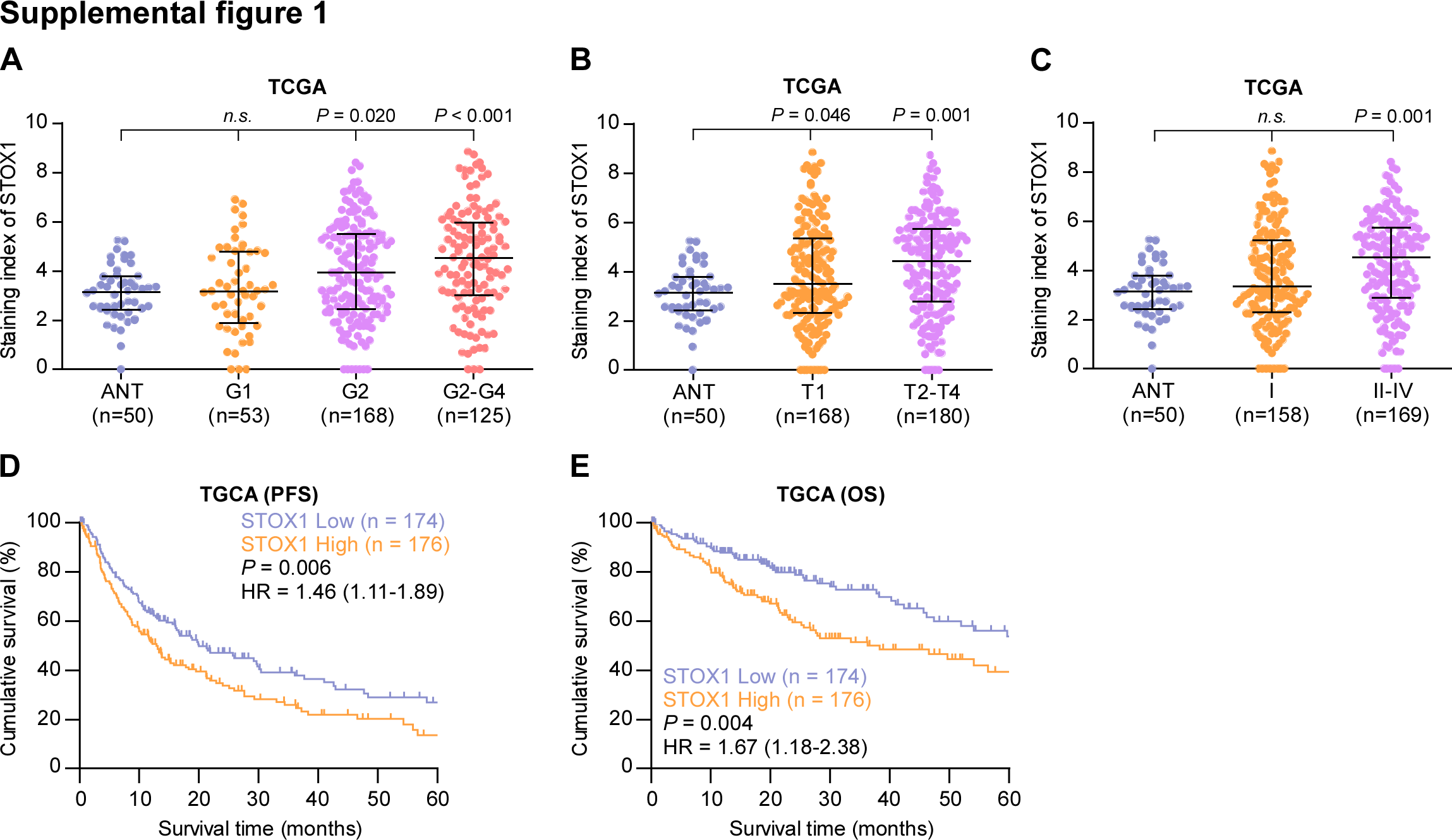


**Supplementary figure 2:**

(A and B) Real-time PCR (A) and Western blot (B) analysis of STOX1-A expression in 7 HCC cell lines, including HepG2, Hep 3B2.1-7, Huh7, Li-7, SNU-182, SNU-387 and SK-Hep-1 and a primary hepatocytes (PHs). GAPDH was used as endogenous controls. *P < 0.05. (C and D) Real-time PCR (C) and Western blot (D) analysis of STOX1-A expression in the indicated HepG2 and Huh 7 cell lines. *P < 0.05.


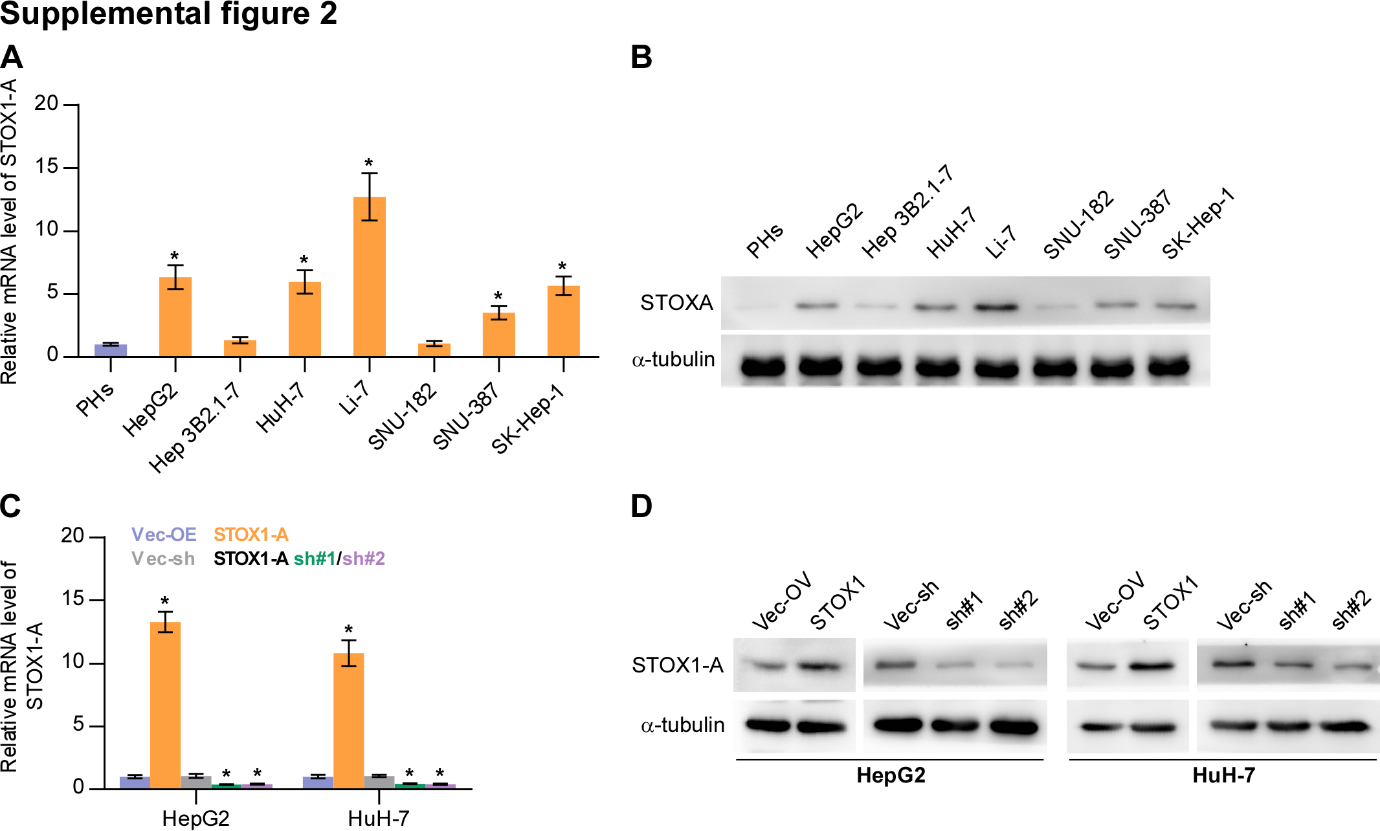


**Supplementary figure 3:**

(A) The effect of Pyrogallol or GSK2795039 on colony-formation ability in STOX1-A overexpressing HCC cells using colony-formation assay.


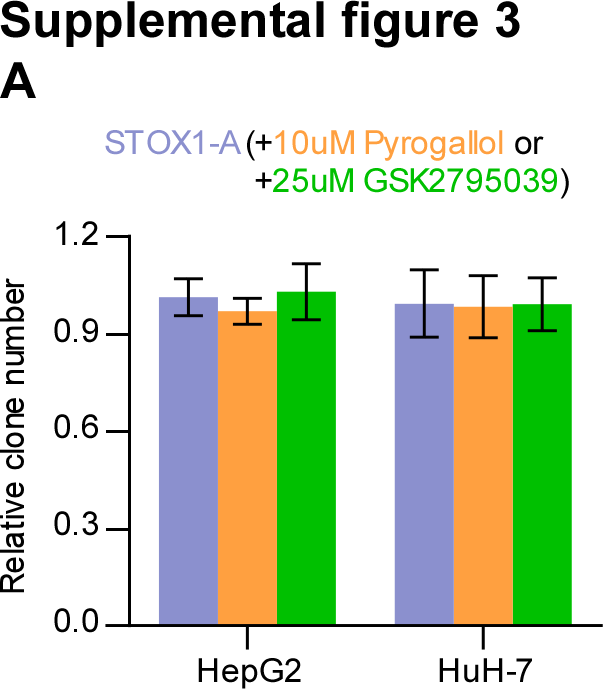


**Supplementary figure 4:**

The expression correlation of STOX-1 with CCNB1 and total AKT in HCC dataset from TCGA.


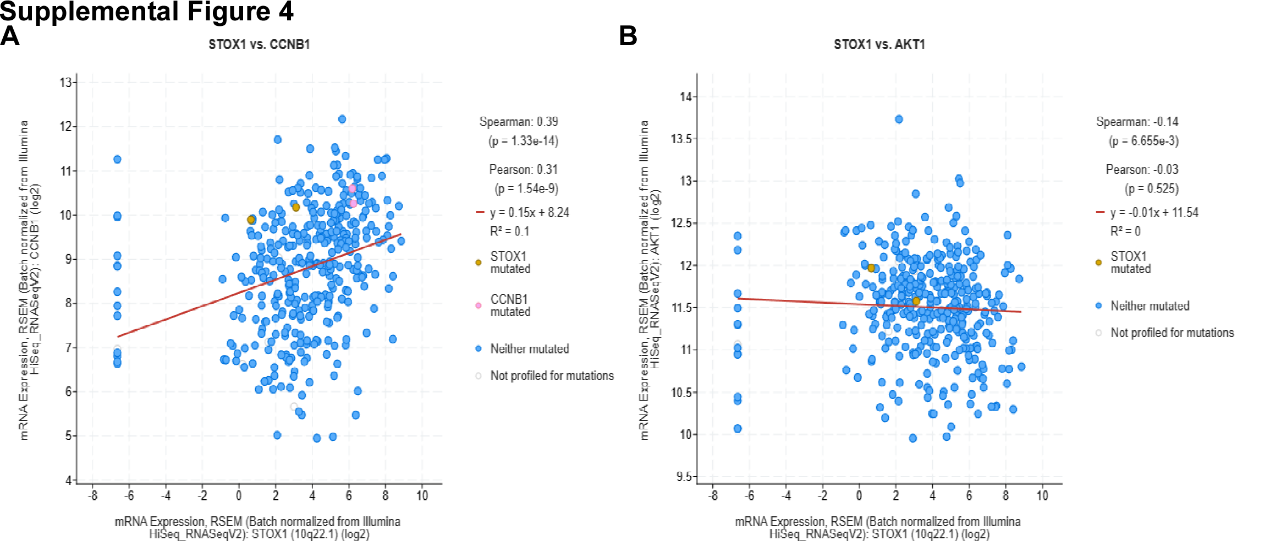


**Supplemental Table 1:**

**The relationship between STOX1 IHC expression level and clinical pathological characteristics in 131 patients with hepatocellular carcinoma.**

| Parameters | Number of cases | STOX1 IHC expression | | P values |
| --- | --- | --- | --- | --- |
|  |  | Low | High |  |
| Age | | | | |
| ≤60 | 95 | 45 | 50 | 0.247 |
| >60 | 36 | 13 | 23 |  |
| Gender | | | | |
| Male | 22 | 9 | 13 | 0.726 |
| Female | 109 | 49 | 60 |  |
| HBV | | | | |
| Positive | 111 | 50 | 61 | 0.676 |
| Negative | 20 | 8 | 12 |  |
| HCV | | | | |
| Positive | 3 | 1 | 2 | 1 |
| Negative | 128 | 57 | 71 |  |
| Grade | | | | |
| G1-G2 | 96 | 52 | 44 | 0.001* |
| G3-G4 | 35 | 6 | 29 |  |
| T classification | | | | |
| T1-T2 | 82 | 39 | 43 | 0.327 |
| T3-T4 | 49 | 19 | 30 |  |
| Stage | | | | |
| Stage I-II | 82 | 39 | 43 | 0.327 |
| Stage III-IV | 49 | 19 | 30 |  |

**Supplemental Table 2:**

**The basic information of 10 patients with hepatocellular carcinoma for STOX1 mRNA and protein expression analysis.**

|  | | Cases (n) | Percentage (%) |
| --- | --- | --- | --- |
| Age | ≤60 | 6 | 60.0 |
|  | >60 | 4 | 40.0 |
| Gender | Male | 10 | 100.0 |
|  | Female | 0 | 0.0 |
| HBV | Positive | 9 | 90.0 |
|  | Negative | 1 | 10.0 |
| HCV | Positive | 1 | 10.0 |
|  | Negative | 9 | 90.0 |
| Grade | G1 | 0 | 0.0 |
|  | G2 | 7 | 70.0 |
|  | G3 | 3 | 30.0 |
|  | G4 | 0 | 0.0 |
| T classification | T1 | 1 | 10.0 |
|  | T2 | 1 | 10.0 |
|  | T3 | 8 | 80.0 |
|  | T4 | 0 | 0.0 |
| N classification | N0 | 9 | 90.0 |
|  | N1 | 1 | 10.0 |
| M classification | M0 | 9 | 90.0 |
|  | M1 | 1 | 10.0 |
| Stage | Stage I | 1 | 10.0 |
|  | Stage II | 1 | 10.0 |
|  | Stage III | 7 | 70.0 |
|  | Stage IV | 1 | 10.0 |

**Supplemental Table 3:**

**The basic information of 41 patients with benign for STOX1 immunohistochemical staining analysis.**

|  | | Cases (n) | Percentage (%) |
| --- | --- | --- | --- |
| Age | ≤60 | 24 | 58.5 |
|  | >60 | 17 | 41.5 |
| Gender | Male | 18 | 43.9 |
|  | Female | 23 | 56.1 |
| Pathological type | Hemangioma | 7 | 17.1 |
|  | Hepatolith | 29 | 70.7 |
|  | Hepatorrhagia | 2 | 4.9 |
|  | Simple cyst | 3 | 7.3 |

**Supplemental Table 4:**

**The basic information of 131 patients with hepatocellular carcinoma for STOX1 immunohistochemical staining analysis.**

|  | | Cases (n) | Percentage (%) |
| --- | --- | --- | --- |
| Age | ≤60 | 95 | 72.5 |
|  | >60 | 36 | 27.5 |
| Gender | Male | 22 | 16.8 |
|  | Female | 109 | 83.2 |
| HBV | Positive | 111 | 84.7 |
|  | Negative | 20 | 15.3 |
| HCV | Positive | 3 | 2.3 |
|  | Negative | 128 | 97.7 |
| Grade | G1 | 20 | 15.3 |
|  | G2 | 76 | 58.0 |
|  | G3 | 33 | 25.2 |
|  | G4 | 2 | 1.5 |
| T classification | T1 | 17 | 13.0 |
|  | T2 | 65 | 49.6 |
|  | T3 | 49 | 37.4 |
|  | T4 | 0 | 0.0 |
| N classification | N0 | 130 | 99.2 |
|  | N1 | 1 | 0.8 |
| M classification | M0 | 129 | 98.5 |
|  | M1 | 2 | 1.5 |
| Stage | Stage I | 17 | 13.0 |
|  | Stage II | 65 | 49.6 |
|  | Stage III | 47 | 35.9 |
|  | Stage IV | 2 | 1.5 |

**Supplemental Table 5:**

**A list of primers used in the reactions for clone PCR.**

| Gene | Sequence (5` – 3`) |
| --- | --- |
| shSTOX1-1#-Forward | CCGGGCAGTCAACCACTCACATCTACTCGAGTAGATGTGAGTGGTTGACTGCTTTTTG |
| shSTOX1-1#-Reverse | AATTCAAAAAGCAGTCAACCACTCACATCTACTCGAGTAGATGTGAGTGGTTGACTGC |
| shSTOX1-2#-Forward | CCGGGATGTCTTTCCAGTGCAAATGCTCGAGCATTTGCACTGGAAAGACATCTTTTTG |
| shSTOX1-2#-Reverse | AATTCAAAAAGATGTCTTTCCAGTGCAAATGCTCGAGCATTTGCACTGGAAAGACATC |
| STOX1-Forward | ATGGCCCGGCCCGTGCAG |
| STOX1-Reverse | TTAAACGTTTATGACTGGAGTTAGCGG |

**Supplemental Table 6:**

**A list of primers used in the reactions for real-time RT-qPCR.**

| Gene | Sequence (5`– 3`) |
| --- | --- |
| STOX1-A-Forward | GTGGAATAGATTCTCCACGGAC |
| STOX1-A -Reverse | TGAAAGATTCTGTAATAATGCTCTACC |
| GAPDH-Forward | TCCTCTGACTTCAACAGCGACAC |
| GAPDH-Reverse | CACCCTGTTGCTGTAGCCAAATTC |
| CCNB1-Forward | AATAAGGCGAAGATCAACATGGC |
| CCNB1-Reverse | TTTGTTACCAATGTCCCCAAGAG |
